# Diagnosis by Volatile Organic Compounds in Exhaled Breath from Patients with Gastric and Colorectal Cancers

**DOI:** 10.3390/ijms24010129

**Published:** 2022-12-21

**Authors:** Jinwook Chung, Salima Akter, Sunhee Han, Yoonhwa Shin, Tae Gyu Choi, Insug Kang, Sung Soo Kim

**Affiliations:** 1 Biomedical Science Institute, Kyung Hee University, Seoul 02447, Republic of Korea; 2 Department of Biochemistry and Molecular Biology, School of Medicine, Kyung Hee University, Seoul 02447, Republic of Korea; 3 Department of Biomedical Science, Graduate School, Kyung Hee University, Seoul 02447, Republic of Korea

**Keywords:** volatile organic compounds, early diagnosis, mass spectroscopy, gastric and colorectal cancer

## Abstract

One in three cancer deaths worldwide are caused by gastric and colorectal cancer malignancies. Although the incidence and fatality rates differ significantly from country to country, the rates of these cancers in East Asian nations such as South Korea and Japan have been increasing each year. Above all, the biggest danger of this disease is how challenging it is to recognize in its early stages. Moreover, most patients with these cancers do not present with any disease symptoms before receiving a definitive diagnosis. Currently, volatile organic compounds (VOCs) are being used for the early prediction of several other diseases, and research has been carried out on these applications. Exhaled VOCs from patients possess remarkable potential as novel biomarkers, and their analysis could be transformative in the prevention and early diagnosis of colon and stomach cancers. VOCs have been spotlighted in recent studies due to their ease of use. Diagnosis on the basis of patient VOC analysis takes less time than methods using gas chromatography, and results in the literature demonstrate that it is possible to determine whether a patient has certain diseases by using organic compounds in their breath as indicators. This study describes how VOCs can be used to precisely detect cancers; as more data are accumulated, the accuracy of this method will increase, and it can be applied in more fields.

## 1. Introduction

The incidence and mortality rate of gastric and colorectal cancer are increasing in East Asia [1,2]. By admission, most patients have already missed the appropriate time for early diagnosis [3,4]. Additionally, the design and establishment of medical systems in developing countries is challenging due to poor financial support from governments [5,6].

Although there are many methods for cancer diagnosis, existing invasive methods such as endoscopy and blood tests impose a heavy burden on patients [7,8]. For instance, patients with gastric and colorectal cancers need to regularly undergo endoscopic examination of the stomach and intestine, respectively [9,10]. To address this problem, a variety of new biomarkers have been designed [7,11].

In recent studies, researchers studying patients’ exhalations with the aim of effectively diagnosing disease have had some success [12,13]. To apply this technique in cancer diagnosis, it is necessary to compare the standardized exhalation parameters obtained from healthy individuals with those sampled from patients [14,15]. This method involves the chemical evaluation of exhaled air and the identification and quantification of compounds such as aldehydes and ketones [16]. It is possible to rapidly analyze the obtained organic matter using analytical chemical assays [17,18]. More studies are required in order to accumulate a large database, with the ultimate goal of significantly lowering mortality rates. This review highlights the importance of mass spectroscopy as a tool to analyze VOCs in the diagnosis of patients with gastric and colorectal cancer.

## 2. Gastric and Colorectal Cancer

This paper addresses in detail gastric and colorectal cancers, which belong to the class of gastrointestinal (GI) cancers that includes all cancers of the organs of the digestive tract [19,20]. GI cancer stem cells (CSCs) are resistant to conventional therapies such as chemotherapy and radiotherapy, and GI cancers are the most lethal and common types of cancer worldwide [21]. There are geographical factors influencing their incidence, and these cancers are especially prevalent in most East Asian countries [22,23]. Furthermore, over one million new cases of these cancers are diagnosed every year worldwide [24,25].

### 2.1. Gastric Cancer

Gastric cancer (GC) is third most common cause of cancer death worldwide [26]. Representing over 90% of GC cases, adenocarcinomas are growths of malignant cells within the lining of the stomach [27]. In the upper digestive system, including the esophagus and stomach, normal tissues can grow in a disordered fashion into carcinoid tumors [28]. The process happens slowly, and is more likely to occur with increasing age [29]. As this disease shows no symptoms in its early stages, it is often diagnosed after it metastasizes to other organs [30].

Depending on the section of the stomach in which the cancer first develops, symptoms tend to progress differently; stomach cancers can be divided into four major categories on this basis: adenocarcinomas, GI stromal tumors, neuroendocrine tumors, and lymphomas [31]. Adenocarcinomas are the most common among these, and there are two major types: intestinal and diffuse [32,33]. The former have a better patient prognosis, whereas the latter are normally discovered submucosally, tend to spread out, and are extremely difficult to detect [34,35,36]. There are also other GCs, but they are extremely infrequent [37].

### 2.2. Colorectal Cancer

Colorectal cancer (CRC) is also divided into colon or rectal cancer, depending on the area in which the cancer first develops [38]. As they have much in common, these cancers are frequently classified together [38,39]. Most colorectal tumors grow from small clumps called polyps on the inner lining of the colon or rectum [40,41]. Polyps have differing tendencies to transform into cancer depending on their type, and not all polyps result in cancer [42,43,44]. Additionally, it takes several years for polyps to develop into cancer [45].

Polyps can be classified into adenomatous, hyperplastic, inflammatory, and sessile serrated varieties [46,47]. Hyperplastic and inflammatory polyps are commonly found, and the adenomatous variety is precancerous [43,48]. Additionally, sessile serrated polyps are often considered adenomas, as they have a higher risk of developing into CRC [49,50]. If the discovered polyps include the following factors, they are at risk of developing into cancer: larger than 1 cm, greater than three in number, and dysplasia discovery after polyp removal [51,52,53]. As time goes by, precancerous polyps develop into cancer in the colon or rectum wall [54,55]. Given that most CRCs are adenocarcinomas (similar to gastric cancers), the cancers originate in cells inside the inner layer which produce mucus as lubrication to protect the colon and rectum [56]. Signet ring cell and mucinous cancers may be associated with a worse prognosis than other adenocarcinomas [57,58,59]. There are also other less common CRCs, such as carcinoid and GI tumors, lymphomas, and sarcomas [60,61].

Although there are differences between these cancer types, patients may not experience any symptoms before cancer diagnosis is made at the early stage or while developing to a later stage [62,63,64,65]. Therefore, early detection, diagnosis, and staging using biomarkers are critical to cancer treatment [66,67].

## 3. What Are Biomarkers?

Biomarkers are indicators that can be used to detect alterations in the body, for example, proteins, DNA, RNA as well as VOCs [68,69,70]. The biomarkers can be used to differentiate between normal and pathological conditions, predict treatment response, and enable objective measurement in the case of a specific disease or cancer [71]. The following conditions should be met, objectively measured, and evaluated in order for an indicator to be defined as a biomarker: normal biological process, disease progression, and drug responsiveness to treatment methods [72].

Biomarkers can be classified into two main groups—invasive or not (Table 1). For example, biomarkers requiring examination of patient body fluids such as blood and serum [73] are invasive, whereas noninvasive sources include breath, urine, and feces [74,75,76]. Additionally, invasive biomarkers can be substituted by analyzing a headspace gas and its medium from a cultured cell line in vitro [77,78]. Further studies on biomarkers for use in disease diagnosis, particularly cancers, are constantly being conducted and expanded to apply their scope in clinical practice [79].

## 4. Breathomics

Breathomics has been a center of research attention since Linus Pauling revealed a complex mixture of an estimated 250 VOCs in human breath [110]. Typical examinations for cancer are based on imaging and blood analysis [111,112]. Computed tomography, for instance, physically and financially burdens patients because of radiation exposure and expense [113]. Thus, breathomics using VOCs obtained from exhaled breath samples is generating a great deal of interest [114].

In 2021, Tsou et al. demonstrated and generalized the concept of how VOCs obtained from patients with cancer could work as biomarkers compared with other noninvasive biomarkers [113]. Although most conventional detection methods have high sensitivity, there are several limitations to these analyses, such as the need for specialized facilities and the financial burden [115,116]. On the other hand, the method of using exhaled breath has numerous advantages, such as high sensitivity, simplicity, and low cost [117,118,119].

Most tests for gastric and colorectal cancers are similar, although there are slight differences [120]. Patients may be reluctant to agree to invasive medical tests such as gastroscopy [121,122]. Therefore, if they are proven to be feasible and valuable for clinical use, health technologies will continue to develop a variety of biomarkers using VOCs from patients’ exhaled breath to relieve this psychological burden [123].

## 5. Methods for VOC Measurement

The instruments used to analyze patients’ exhaled breath include gas chromatography–mass spectroscopy (GC–MS), collection tools such as the Tenax TA (pipe), and Tedlar bags for sample storage [124,125]. The pipes are especially useful for storage of low concentrations of exhaled gas because they contain a solid absorbent [126,127]. In brief, for the analysis process, a subject suspected of having a disease breathes into a Tedlar bag through a pipe [128]. Next, the collected sample is analyzed using GC–MS and the patient’s VOC profile is compared with VOC profiles obtained from healthy individuals [129,130,131]. It is critical that atmospheric VOCs are also collected in other tubes in order to know in advance the variables that may affect the experiment [113]. Various other analytical instruments have also been used, such as ion mobility spectrometry, selected ion flow tube–mass spectrometry (SIFT-MS), proton transfer reaction–mass spectrometry (PTR-MS), and comprehensive 2D gas chromatography [132].

SIFT-MS, which learns numerous data using extreme gradient boosting (XGBoost), is a point of convergence between specific and reliable quantification, and it is much sought after [133,134,135,136]. In other words, SIFT-MS combined with big data is useful for the qualitative analysis of VOCs in real time [136]. Before everything else, the tool classifies subjects based on their physical condition and the result of VOC analyses [137,138].

## 6. Cancer-Related VOCs in Exhaled Breath

Global Cancer Statistics reported that 46% of people worldwide experienced breast, lung, prostate, and gastric and colorectal cancers in 2020 [139] (Table 2). Aldehydes and ketones, which are primarily expressed in all cancers, are discussed in [140]. Although common chemicals such as alcohols and benzenes were also noted, they were considered to be from exogenous factors such as smoking (Table 3). The aim of this work was to clarify the biochemical pathways of aldehydes and ketones in order to determine their origins. The concentration changes in exhaled breath from GI cancer patients can be directly associated with biomarkers of cancer quantification because the metabolic processes of cancer cells produce or reduce abnormal organic compounds compared with normal cells [140,141]. An analysis of the Cancer Odor Database (COD) developed by Janfaza et al. indicates that some VOCs contribute to particular types of cancer and have potential as biomarkers [142,143].

### 6.1. Aldehydes

As indicated in Table 2, aldehydes are associated with all five of the specified types of cancer (breast, lung, colon, prostate, and stomach). Among the aldehydes, hexanal, nonanal, and heptanal aldehydes are commonly detected in patients’ exhaled breath, and in blood, saliva, and urine [143].

Since the composition of the membrane lipids in cancer cells is changed, some saturated and unsaturated lipids are observed at altered levels compared to the profiles associated with normal, healthy individuals [158,159]. Increased concentrations of unsaturated fatty acids might promote the production of some aldehydes through lipid peroxidation [160,161,162]. For this reason, the metabolism of aldehydes in cancer cells differs from that in normal cells [163,164].

#### 6.1.1. Metabolic Pathway of Aldehydes in Cancer and Normal Cells

Ethanol, an alcohol, is oxidated in diverse metabolic mechanisms by enzymes such as aldehyde dehydrogenase (ALDH), alcohol dehydrogenase (ADH), and cytochrome P450 (CYP450) with hydrogen peroxide [165,166,167]. Generally, ethanol is oxidated to acetaldehyde by ADH [168,169]. Then, the aldehyde is degraded to carboxylic acid using ALDH [170,171].

Since metabolic demands are rarely lowered in normal cells, ALDH is not overexpressed to detoxify and lower reactive oxygen species (ROS) production [172]. In contrast, toxic aldehydes and ROS accumulate in cancer tissues [173].

The primary alcohol is typically metabolized in two steps in the liver [174]. First, ethanol is oxidated to acetaldehyde through enzymes such as ADH and cytochrome P2E1 (CYP2E1) [175]. With ALDH, acetaldehyde is additionally metabolized to acetate as a further step [176,177]. Above all, the results of these reactions depend on the enzymes; even acetaldehyde, which is toxic [178,179,180] and carcinogenic, has the potential to accumulate [176]. This accumulation has grave implications for DNA, suppressing DNA repair and damaging the antioxidative defense system (AODS) [181,182].

In the first step of oxidation, various acetaldehydes are generated through ADH1B and ADH1C, which belong to the same subfamily [183,184,185]. However, toxic acetaldehydes, which are the result of oxidation by CYP2E1 [167,186], produce ROS [174,187] and damage AODS [188]. As a result, insufficiently detoxified ROS cause the formation of DNA adducts [189,190].

**Figure 1:**
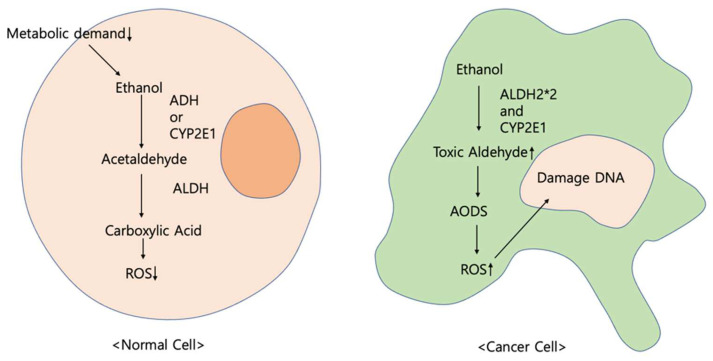
Complete oxidation mechanism.

In the second step, acetaldehyde is degraded by ALDH [166,191]. The enzyme has polymorphisms, such as ALDH2*1 and ALDH2*2 [188]. Among these, ALDH2*2 contributes to the accumulation of acetaldehyde [192,193,194] (Figure 1).

#### 6.1.2. Cytochrome P450 and Reactive Oxygen Species

- Cytochrome P450 (CYP450)

As a coenzyme containing heme, CYP450 is a multigenic family of proteins [195,196]. Most of these enzymes are responsible for different enzymatic reactions and are well known as electron transport oxidases [197,198]. Above all, CPY450 plays a key role in diverse metabolism and detoxification processes [199]. Moreover, the enzyme is involved in miscellaneous enzymatic reactions such as fatty acid metabolism [200,201]. CYP450 is primarily found within the endoplasmic reticulum, and in mitochondria in the liver [202]. CYP450 is classified based on electron transport proteins, for instance, microsomal and mitochondrial [203].

ROS production is closely related to CYP450 [204]. CYP450 enzymes, which can control carcinogenic activity, are involved in cancer initiation and promotion [205,206]. Furthermore, when CYP450s are overexpressed in a tumor cell, ROS are manufactured by the coenzymes [207,208]. Among the subfamily of CYP450 enzymes, CYP2E1 is mainly correlated with ROS production [41]. Specifically, the overexpression of CYP2E1 results in a high level of inflammatory cytokines compared to normal cells [209,210].

- Reactive Oxygen Species

Although ROS are signaling molecules for normal cells, ROS generation can cause harm to autophagy, unfolded protein response, and several cellular organelles, with the potential to lead to disorder in normal cell viability [211,212]. For that reason, unnecessary ROS should be eradicated in order to maintain redox homeostasis [171].

Normal cells have enough adaptive ability to protect themselves from the adverse influences of ROS [213]. In contrast, where there is anomalous ROS production, redox imbalance can provoke advancement to the initiation and development of several cancer types. Additionally, the metabolism of cancer tumors generates high ROS concentrations [214].

At low ROS levels, biological processes of cancer cells such as development and survival are limited because cells have the capability of antioxidant activity to repair damage [215,216,217]. At high ROS concentrations, cellular organelles are damaged, and the DNA repair pathway is disrupted [218,219,220]. Additionally, increased oxidative stress results in a high rate of aldehyde production [143].

### 6.2. Ketones

Similarly to aldehyde, ketones are derived from and affected by external factors such as diet [221]. Nevertheless, in many cancers, the production of ketones begins from a typical mechanism of increasing long-chain fatty acid (LCFA) oxidation to increase the ketone body production in the mitochondria of the liver [222,223]. As the first step in the catabolism of fatty acids, β-oxidation breaks down fatty acids using electron transport chain factors such as NADH and FADH_2_, and produces acetoacetyl-CoA (acac-CoA) [224,225].

Normal and tumor tissues regulate ketone bodies differently [226,227]. In normal tissue, ketone bodies regulate cellular energy supply from glucose to fatty acids and ketones to regulate blood glucose, since glucose provision is restricted by 3-hydroxy-3-methlglutaryl-CoA synthase 2 (HMGCS2) and solute carrier family 16 (SLC16A6) [228,229]. Additionally, ketone bodies can be degraded into acetyl-CoA to enter the tricarboxylic acid (TCA) cycle, which produces energy and enhances cell viability [230,231,232].

The mitochondrial structure of cancer cells is different to that of normal cells; ketone bodies may increase their oxidative stress via the TCA cycle [228]. Moreover, electrons are overproduced by NADH and ${\mathrm{FADH}}_{2}$ in the TCA cycle and β-oxidation, and are moved into the mitochondria of cancer cells [232]. Additionally, the antioxidant system pathway is inhibited because of increased ROS and causes oxidative stress damage in low-carbohydrate conditions [215,233].

**Figure 2:**
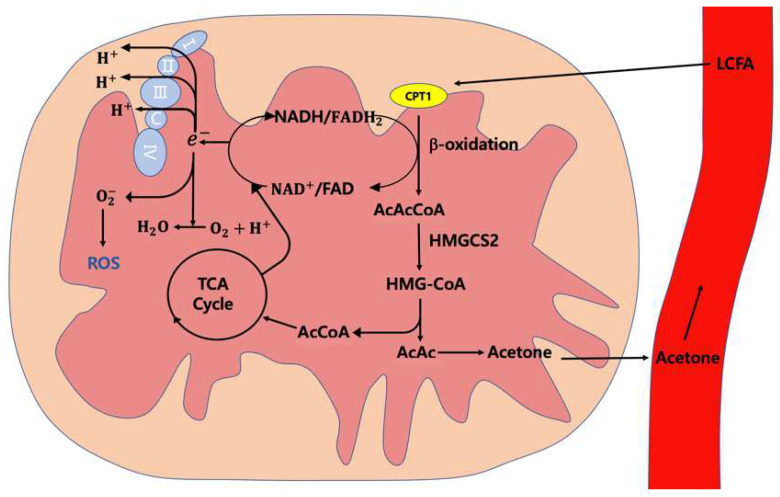
Cancer cell ketogenesis.

Acetoacetyl (AcAc) directly results in the formation of ketone bodies, which are released into the plasma [28,234,235]. As these ketones are weakly soluble, they are transported through blood vessels to the lungs and are then exhaled [236] (Figure 2).

Acetone is the smallest ketone, and it is continuously produced during acetoacetate decarboxylation [237,238] even after being degraded into acetol by CYP2E1 [239]. There are different mechanisms involved in ketone production. For example, 2-nonanone is generated via nonane metabolism by CYP450 [240,241].

There are four ketones that are considered cancer biomarkers: 2-nonanone, 3-heptanone, 4-heptanone, and cyclohexanone [242,243]. Although there are limitations to their use in the detection and investigation of cancers, among these ketones, cyclohexanone is extensively observed in patients with chronic pulmonary disease and not in healthy individuals [244,245].

## 7. Summary and Future Perspectives

### 7.1. Summary

VOCs contain invaluable information about the biochemical metabolization of cancer cells [246]. According to some articles, some compounds are related to specific cancers and can be used to distinguish between patients and healthy people [100]. Aldehyde and ketone can be identified in the breath just minutes after being released from tissues because they are slightly soluble in blood [247].

As reported, 10 VOCs are associated with gastric and colorectal cancers, in addition to aromatics and hydrocarbons from exogenous factors. Although these organic compounds can all be deemed important biomarkers, hexanal and 3-heptanone are especially reported to be closely related to gastric and colorectal cancers according to studies using various methods, although exhaled breath has not been studied in this regard [248,249,250].

According to the other reports, many short-chain fatty acids (SCFAs), such as acetate, have been found at high concentrations in the exhaled breath of patients with colorectal [153] and gastric cancer [156] in comparison to healthy subjects. This result shows that SCFAs in the breath of GC patients might be generated by the metabolism of stomach cancer cells.

### 7.2. Future Perspectives

The studies described herein found significant cancer-related aspects of VOC profiles. In the medical field, biomarkers are a cornerstone of a paradigm shift towards a personalized medical system centered on prevention, with treatment based on experience and statistics beyond the existing collective diagnostic tests [251,252,253]. The global biomarker market is growing steadily [254]. Biomarker research on many diseases is growing alongside the development of the medical industry [71,255]. The development of more advanced biomarkers is in progress, and this is expected to bring more progressive biomarker use [140,256,257,258]. The fatality rates of some cancers are still high, in view of the fact that it is difficult to be aware of symptoms before the disease has developed to a fatal level, despite the use of advanced medical technologies [259]. To make matters worse, high costs make it difficult for patients to access medical tests without insurance [260,261,262].

The metabolization of aldehydes and ketones for gastric and colorectal cancer has been comprehensively reviewed in this article. Moreover, we have demonstrated that VOCs contain invaluable information about the biochemical metabolization of cancer cells. Therefore, the comprehensive analysis of discernible VOCs in patients’ exhaled breath may reduce the burden of invasive medical tests for patients, and may enable the early detection of cancer and the efficient prediction of prognosis following surgery with a small outlay.

Regarding instrumentation, SIFT-MS can be used to analyze considerable quantities of quantitative data with the XGBoost model and to predict cancers based on VOC factors [113,263]. Based on machine learning and deep learning algorithms, this instrument can accurately determine cancer using VOCs from patients’ exhaled breath and reduce the interference of environmental factors, resulting in accurate prediction models [113,264]. As science has advanced, big data associated with research on how VOCs are related to cancers has been accumulating for over fifty years, and thus, it can now be processed [265,266]. If large amounts of data continue to accumulate as additional research continues, further research will be still easier.

In addition to SIFT-MS, bioelectronic and olfactory-receptor-based sensors have shown remarkable sensitivity upon their merging into a primary transducer [267,268,269]. This has many advantages—it is simple to use and sufficiently inexpensive that it can be made available to everyone [132,267,268]. Thus, these are promising alternatives to conventional diagnostic instruments [270].

## 8. Conclusions

The study of VOCs from exhaled breath is an area of significant innovation [271]. It has a great deal of potential to yield biomarkers for GI cancer, although further studies are required because sufficient data have not yet been collected. Above all, the origin of VOCs can include exogenous factors, especially physical activities and smoking, which change the pattern of VOCs [272,273].

For instance, acetone, with an abnormal fruity odor, might be considered an adequate cancer biomarker [239]. However, the chemical cannot itself represent an appropriate biomarker because the acetone concentration in breath changes during activities such as exercising or fasting [274]. Furthermore, there are limitations in that the origins of most VOCs (e.g., 4-heptanone) are unclear [248], and thus, they are not recommended for use as biomarkers [275].

Similarly, there are still limitations to research on the origins of most VOCs [248]. However, analyzing big data with advanced instruments might be useful and helpful in solving the problem of VOCs related to gastric and colorectal cancer. Consequently, there is a possibility that, in the future, we will be able to easily prevent and treat cancer using these revolutionary biomarkers.

## Figures and Tables

**Table 1:** Typical biomarkers and their use.

Type of Biomarker		Characteristics	Cancer	Refs.
**Invasive**	**Blood**	Shows chemicals and proteins originated by cancer cells	Prostate, ovarian, and testicular cancer	[80,81,82,83,84]
	**Endoscopy**	Used to identify idiopathic symptoms and observe prognosis	Gastric and colorectal cancer	[85,86,87,88,89,90,91]
**Noninvasive**	**Nipple aspirate fluid**	Indicates the degree of disease progression and enables early diagnosis	Breast cancer	[92,93]
	**Urine**	Highly sensitive and economical source of biomarkers, allows surveillance of therapeutic result	Prostate, bladder, endometrial, and pancreatic cancer	[94,95,96,97,98,99]
	**Breath**	Accurate detection in a short time, can be used to predict cancers	Lung, breast, gastric, and colorectal cancer	[100,101,102,103,104,105,106,107]
	**Sweat**	Measurable in small quantities and is not limited by consultation space restrictions	Lung cancer	[108,109]

**Table 2:** The ranked cancers related to eight typical VOCs based on references.

Rank	Cancer	Volatile Organic Compounds	Refs.
**1**	Breast	Alkanes, Aldehydes, Esters, Ketones	[119,144]
**2**	Lung	Alcohols, Aldehydes, Ethers	[145,146]
**3**	Colon	Alcohols, Aldehydes, Alkanes, Ketones	[131,147]
**4**	Prostate	Acetones, Alcohols, Aldehyde Ammonias	[148,149]
**5**	Stomach	Alcohols, Aldehydes, Ketones	[150,151]

**Table 3:** The altered VOCs in gastric and colorectal cancer patients.

Cancer		Volatile Organic Compounds	Ref.
**Colon**	**Increased**	Alcohols, Aldehydes (Benzaldehyde), Acetone (Ketones), Indole	[131,140,152,153,154]
	**Decreased**	Benzene Ethyl	[140]
**Stomach**	**Increased**	Alcohols (Phenol, 2-Butoxy-Ethanol), Aldehydes (Benzaldehyde, Propanal), Acetone (Ketones)	[140,150,155,156,157]
	**Decreased**	Pentanoic acid, 1,3,5-Trimethylbenzene	[140]

## Data Availability

Not applicable.

## Abbreviations

VOCs	Volatile Organic Compounds
GI	Gastrointestinal
CSCs	Cancer Stem Cells
GC	Gastric Cancer
CRC	Colorectal Cancer
GC-MS	Gas Chromatography–Mass Spectroscopy
SIFT-MS	Selected Ion Flow Tube–Mass Spectrometry
PTR-MS	Proton Transfer Reaction–Mass Spectrometry
ALDH	Aldehyde Dehydrogenase
ADH	Alcohol Dehydrogenase
CYP450	Cytochrome P450
ROS	Reactive Oxygen Species
CYP2E1	Cytochrome P2E1
AODS	Antioxidative Defense System
LCFA	Long-Chain Fatty Acid
acac-COA	Acetoacetyl-CoA
HMGCS2	3-Hydroxy-3-Methlglutaryl-CoA Synthase 2
SLC16A6	Solute Carrier Family 16
TCA	Tricarboxylic Acid
AcAc	Acetoacetyl
SCFAs	Short-Chain Fatty Acids
